# The British Association

**Published:** 1901-09-14

**Authors:** 


### The Hospital

A Journal of

The Medical Sciences and Hospital Administration.

Vol. XXX.—No. 781.] Edited by Sir Henry Burdett, K.C.B., and Solomon C. Smith, M.D., M.R.C.P. [September 14, 1901.

### The British Association.

The Presidential Address of Professor Rücker, delivered on Wednesday evening before the meeting of the British Association at Glasgow, was of a somewhat more recondite character than has been usual with his predecessors. It was calculated to enable every hearer to depart, after his well-spent hour, saying:—

> "There fixed thoughts to questions did I lend,
> Which hover on the bounds of mortal ken,
> And have perplexed, and will unto the end,
> Perplex the brains of men."

Instead of conducting his audience in a sort of triumphant march along paths through the domain which had been rescued during the year from the territory of ignorance, or which had been newly fertilised by the application of knowledge, instead of passing in review the recent successful labours of science, either in the whole or in a more limited portion of the field of her activity, the professor confined himself to indicating directions in which her very foundations had been assailed, and to pointing out the strength of the positions which, in spite of such assaults, are still occupied by the two bases of all hitherto received physical theories of the universe, the atomic theory, and the undulatory theory of light and of other forms of force, as based upon the conception of an all-pervading ether as a medium of wave movement. The limitations of time, imposed by the conditions under which the Address was delivered, rendered it impossible to deal with the questions referred to in a manner commensurate with their far-reaching importance, or even to sketch the evidence by which any of the doubts thrown upon accepted beliefs can be supported, and the Professor was forced to content himself with the almost barren statement that Professor Poincaré, addressing a congress of physicists in Paris, and Professor Poynting, addressing the physical section of the Association, have recently discussed the true meaning of our scientific methods of interpretation, that Dr. James Ward has lately delivered an attack of great power on many positions which eminent scientific men have occupied, that the approaching end of the nineteenth century led Professor Hæckel to define in a more popular manner his own very definite views as to the solution of the "Riddle of the Universe," and that a great school of chemists, building upon the thermodynamics of Willard Gibbs and the intuition of Van t'Hoff, have shown with wonderful skill that, if a sufficient number of data of experiment are assumed, it is possible, by the aid of thermodynamics, to trace the form of the relations between many physical and chemical phenomena without the help of the atomic theory.

Notwithstanding the position, apparently remote from practicality, which the new doctrines thus briefly indicated must for the present be admitted to occupy, it is none the less true that everything which bears either upon the constitution of matter, or upon the relations between matter and the forces by which it is influenced or controlled, must at no distant period of time be brought into relation with the changes in the human body which in their normal occurrence constitute health, and which, when injuriously modified, either by internal or external agencies, present themselves to our observation as disease. Vast as are the potentialities of physical science in relation to the externals of life, or in an infinite number of applications to arts, manufactures, locomotion, and analogous purposes, they are still more vast in their relations to the integrity of the human machine itself, and to the means by which that integrity may be preserved; and hence it follows that, as in the well-known Terentian line, nothing which appertains to physical science can ever be foreign to the province and the pursuits of the physician. Our bodies represent matter in its highest and most complete state of organisation; and every advance in knowledge which can throw light upon that organisation, either in ourselves or in simpler forms of life, will be necessarily contributory to the attainment of the highest end of all knowledge, the knowledge of ourselves and of our place in nature, and of the means by which that place may either be elevated or rendered more secure. Inasmuch as it is repellent to the human mind to return to the alphabet after fancying that it has made some small progress in the language, it is in a certain sense satisfactory to perceive that Professor Rücker is not at present disposed to exchange old lamps for new ones, and that he stoutly defends the atomic theory, showing the large number of phenomena in relation to which no effective substitute for it has hitherto been advanced. It may be granted, he says, that we have not yet framed a consistent image either of the nature of the atoms or of the ether in which they exist, but, in spite of this, and of the consequently tentative nature of some of the dependent theories, he believes that the atomic theory unifies so many facts, and simplifies so much that is complicated, as to give us a right to insist, at all events until an equally intelligible rival hypothesis is produced, that the main structure of our theory is true, and that atoms are not merely helps to puzzled mathematicians, but that they exist as veritable and all-pervading physical realities.

